# Clearance of senescent cells with ABT-263 improves biological functions of synovial mesenchymal stem cells from osteoarthritis patients

**DOI:** 10.1186/s13287-022-02901-4

**Published:** 2022-06-03

**Authors:** Yugo Miura, Kentaro Endo, Keiichiro Komori, Ichiro Sekiya

**Affiliations:** Center for Stem Cell and Regenerative Medicine, Tokyo Medical and Dental University (TMDU), 1-5-45 Yushima, Bunkyo-ku, Tokyo, 113-8510 Japan

**Keywords:** Mesenchymal stem cells, Senescence, Senolytic drug, Osteoarthritis, ABT-263, Synovium

## Abstract

**Background:**

Osteoarthritis (OA) is an age-related joint disease characterized by progressive cartilage loss. Synovial mesenchymal stem cells (MSCs) are anticipated as a cell source for OA treatment; however, synovial MSC preparations isolated from OA patients contain many senescent cells that inhibit cartilage regeneration through their senescence-associated secretory phenotype (SASP) and poor chondrogenic capacity. The aim of this study was to improve the biological function of OA synovial MSCs by removing senescent cells using the senolytic drug ABT-263.

**Methods:**

We pretreated synovial MSCs derived from 5 OA patients with ABT-263 for 24 h and then evaluated senescence-associated beta-galactosidase (SA-β-gal) activity, B cell lymphoma 2 (BCL-2) activity, apoptosis, surface antigen expression, colony formation ability, and multipotency.

**Results:**

The ABT-263 pretreatment significantly decreased the percentage of SA-β-gal-positive cells and BCL-2 expression and induced early- and late-stage apoptosis. Cleaved caspase-3 was expressed in SA-β-gal-positive cells. The pretreated MSCs formed greater numbers of colonies with larger diameters. The expression rate of CD34 was decreased in the pretreated cells. Differentiation assays revealed that ABT-263 pretreatment enhanced the adipogenic and chondrogenic capabilities of OA synovial MSCs. In chondrogenesis, the pretreated cells produced greater amounts of glycosaminoglycan and type II collagen and showed lower expression of senescence markers (p16 and p21) and SASP factors (MMP-13 and IL-6) and smaller amounts of type I collagen.

**Conclusion:**

Pretreatment of synovial MSCs from OA patients with ABT-263 can improve the function of the cells by selectively eliminating senescent cells. These findings indicate that ABT-263 could hold promise for the development of effective cell-based OA therapy.

**Supplementary Information:**

The online version contains supplementary material available at 10.1186/s13287-022-02901-4.

## Background

Knee osteoarthritis (OA) is the most common joint disease worldwide. It is characterized by a progressive loss of articular cartilage and meniscus degeneration associated with aging and synovitis [1] and has recently been designated an age-related inflammatory disease [2]. OA patients suffer from a variety of symptoms (e.g., pain, stiffness, swelling, and loss of mobility), so the recommended therapies can range from pain control to surgery [3]. However, none of the available treatments can improve cartilage loss sufficiently to affect patients fundamentally. New treatments are therefore needed to overcome this limited effectiveness, because the number of OA patients is anticipated to increase in today’s aging society.

One promising OA treatment that has emerged in the last two decades is the use of mesenchymal stem cells (MSCs). These cells are considered a viable therapeutic alternative for OA due to their pleiotropic abilities, such as self-renewal, multipotency, and secretion of cytokines and growth factors [4–8]. MSCs can be obtained from various tissues, but synovial MSCs have a higher colony formation ability and chondrogenic potential than MSCs from other tissues, and are therefore promising for treating damaged cartilage [9–12]. We previously demonstrated that synovial MSCs transplanted into rabbit cartilage or meniscus defects could promote regeneration by producing abundant cartilage matrices [13, 14]. Similarly, intra-articular injection of synovial MSCs suppressed the progression of OA in rats [15]. Our recent clinical study in human OA patients showed that transplantation of autologous synovial MSCs improved the clinical outcomes and the amounts of cartilage that could be assessed by automatic magnetic resonance imaging (MRI) [16].

One issue with the use of MSCs as an OA treatment is that the MSC preparations typically contain senescent cells. Senescence in cells, including MSCs, is triggered by some type of stress that causes the activation of the p16 or p21 pathways. This puts the cells into permanent cell-cycle arrest, resulting in resistance to apoptosis [17]. The senescent cells remain metabolically active and release factors referred to as senescence-associated secretory phenotype (SASP) factors, which include proinflammatory cytokines (e.g., interleukin-6; IL-6) and proteinases (e.g., matrix metalloprotease-13; MMP-13) [17]. SASP plays a crucial role in accelerating the senescence of other neighboring cells and deteriorating their function [17].

In OA knees, chondrocyte senescence can be induced by a number of different stresses [1, 18], and recent reports indicate that senescent cells also accumulate in the OA synovium and in the MSCs isolated from it [19, 20]. This means that senescent cells are also present in cultures of synovial MSCs that are used as OA therapeutics. The cultures containing senescent MSCs lack full OA therapeutic efficacy due to their release of SASP factors and their poor chondrogenic capacity [20]. Therefore, clearance of senescent cells from MSC cultures intended for therapeutic use is necessary to maximize the efficacy of OA synovial MSCs.

Senescent cells can be selectively removed from cultures by drugs known as senolytics [18, 21]. Several senolytics have been introduced in previous reports. The first senolytic drugs were dasatinib, a tyrosine kinase inhibitor, and quercetin, a PI3K/Akt inhibitor [18, 21, 22]. The combination of dasatinib and quercetin can result in the elimination of senescent cells from various tissues [21, 23]. ABT-263 (Navitoclax) is also one of the best studied senolytic drugs [18, 22, 24]. ABT-263 is an inhibitor of the anti-apoptotic proteins of B cell lymphoma 2 (BCL-2) protein family, and its suppression of BCL-2 activity can induce senescent cells in various tissues to undergo apoptosis [25]. Grezella et al. demonstrated that ABT-263 exerted a senolytic effect in replicative senescent human bone marrow-derived MSCs [26]. However, no studies have yet focused on the use of ABT-263 for the qualitative alteration of MSCs, and little is known about the effects of this drug on senescent OA synovial MSCs.

Our aim in the present study was to use ABT-263 to selectively eliminate senescent cells from synovial MSC samples derived from patients with OA as a way to improve the quality of MSCs used for OA therapy. We pretreated OA synovial MSCs with ABT-263 for a short period and then evaluated the extent of senescent cell clearance and the in vitro biological potencies of the treated MSC samples.

## Methods

### Isolation of human synovial MSCs

Human synovial samples were acquired from 5 OA donors (age range 71–80 years; one male and four females; Kellgren–Lawrence grade 3 or 4) who underwent total knee arthroplasty. The synovium was digested with 3 mg/mL *Clostridium histolyticum*-derived collagenase type V (Sigma-Aldrich, Saint Louis, MO, USA) at 37 °C for 3 h. Debris was then removed by passing the digest through a 70 µm cell strainer (Greiner Bio-One, Kremsmünster, Austria), and the filtrate was collected. The cells were washed with phosphate-buffered saline (PBS), and the nucleated cells were counted and plated in 145 cm^2^ dishes at a density of 2000 cells/cm^2^ in a growth medium consisting of α-modified essential medium (α-MEM; Thermo Fisher Scientific, Waltham, MA, USA) and 10% fetal bovine serum (Thermo Fisher Scientific), and 1% antibiotic–antimycotic (Thermo Fisher Scientific). After 2 weeks of culture, the cells were detached with 0.25% trypsin and 1 mM EDTA (Thermo Fisher Scientific) and then cryopreserved in growth medium supplemented with 5% dimethyl sulfoxide (DMSO; Wako, Tokyo, Japan).

### Pretreatment with ABT-263

**Fig. 1:**
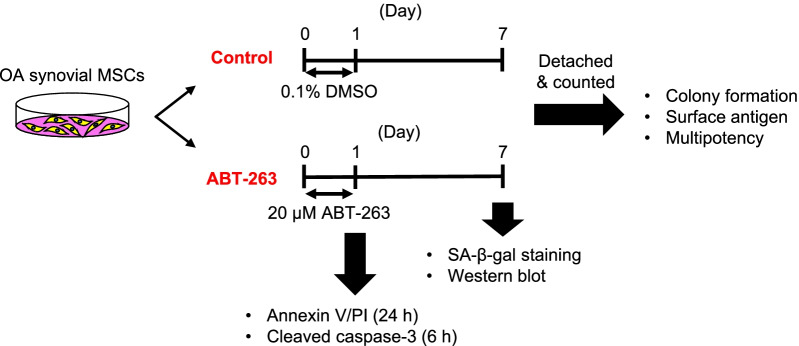
Scheme of the experiments. Synovial mesenchymal stem cells (MSCs) from osteoarthritis (OA) patients were treated with 0.1% dimethyl sulfoxide (DMSO) or 20 µM ABT-263 in DMSO for 24 h. After the treatment, the cells were expanded for another 6 days and used for the experiments. Annexin V/propidium iodide (PI) staining and cleaved caspase-3 immunostaining were performed immediately after the treatment for 24 and 6 h, respectively

ABT-263 was obtained from MedChemExpress (Monmouth Junction, NJ, USA). We plated human synovial MSCs at passage 1 in 145 cm^2^ dishes (Thermo Fisher Scientific) at a density of 2000 cells/cm^2^ and cultured them in 20 mL of growth medium. The cells were then treated for 24 h with 0.1% DMSO (control group) or with 20 µM ABT-263 in DMSO (ABT-263 group). After the treatment, the culture medium was changed to the growth medium, and the cells were further expanded for 6 days. Apoptosis assays were performed immediately after the treatment. An experimental scheme is shown in Fig. 1.

### Senescence-associated β-galactosidase (SA-β-gal) staining

SA-β-gal staining was performed using a Senescence β-Galactosidase Staining Kit (Cell Signaling Technology, Danvers, MA, USA), according to the manufacturer’s instructions. Briefly, the cells were fixed with fixative solution and then incubated at 37 °C in staining solution at pH 6.0 for 16 h. Senescent cells were identified as blue-stained cells under brightfield microscopy. Cells positive for SA-β-gal staining were counted manually in four fields at × 10 magnification.

### Apoptosis assay

Human synovial MSCs were treated with 0.1% DMSO or 20 µM ABT-263 for a day. After the treatment, the cells were detached with trypsin, suspended in PBS, and the cell suspension was incubated with FITC-annexin V and propidium iodide (PI) using an FITC-Annexin V Apoptosis Detection Kit (BD Biosciences, NJ, USA). The fluorescence intensity was evaluated using a FACSVerse II system (BD Biosciences). The obtained data were analyzed using FlowJo version 8.7.1 (Ashland, OR, USA).

### Co-staining of SA-β-gal and cleaved caspase-3

Human synovial MSCs were cultured in 8-well chamber slides (Thermo Fisher Scientific). The cells were treated for 6 h with ABT-263 and then stained for SA-β-gal. The cells were fixed with 4% paraformaldehyde (Sigma-Aldrich), permeabilized, and blocked with 0.3% Triton and 5% normal goat serum. The cells were then incubated overnight at 4 °C with antibody against cleaved caspase-3 (1:400; Cell Signaling Technology). After three washes with PBS, the cells were incubated with anti-rabbit secondary antibodies with horseradish peroxidase (1:200; Abcam, Cambridge, UK) for 1 h at room temperature. Diaminobenzidine (DAB) solution (Dako North America, Carpinteria, CA, USA) was applied for 10 min.

### Western blot analysis

Protein was extracted from cells pretreated in RIPA buffer with or without ABT-263 (Thermo Fisher Scientific). The protein concentration was calculated using a Pierce BCA Protein Assay Kit (Thermo Fisher Scientific). Protein samples (10 µg) were then loaded onto Mini-PROTEAN TGX Precast Gels (BIO-RAD) and separated by electrophoresis. The separated proteins were transferred to Trans-Blot Turbo Mini PVDF membranes (BIO-RAD), and the membranes were blocked with 5% skim milk for 1 h. The membranes were then incubated with the primary antibodies for BCL-2 (1:1000; Cell Signaling Technology) and β-actin (1:1000; Cell Signaling Technology) overnight at 4 °C, washed with Tris-buffered saline containing 0.05% Tween-20, and then incubated with the secondary antibodies conjugated with horseradish peroxidase (1:5000; Abcam) for 1 h at room temperature. SuperSignal West Femto Maximum Sensitivity Substrate (Thermo Fisher Scientific) was then applied to the blot. Chemiluminescence was detected by ChemiDoc XRS + system (BIO-RAD) and analyzed by Image Lab 6.1 (BIO-RAD). The integrated intensity of BCL-2 was normalized to that of β-actin.

### Colony-forming assays

Cells pretreated with ABT-263 were seeded at 100 cells/dish in 145 cm^2^ dishes (Thermo Fisher Scientific) and cultured for 2 weeks. The cells were then fixed with 10% neutral buffered formalin (Muto Pure Chemicals, Tokyo, Japan), followed by staining with 0.5% crystal violet (Wako). Colonies were counted manually, and the diameter was assessed using Fiji/Image J (National Institute of Health, Bethesda, MD, USA) and the “Analyze Particles” command. Colonies less than 2 mm in diameter and faintly stained colonies were ignored.

### Surface antigens

Human synovial MSCs were detached with trypsin and suspended in PBS containing 2% FBS and 5 mM EDTA. The MSCs were then stained for 30 min at 4 °C with CD44-APCH7, CD73-V450, CD90-PE-Cy7, CD105-APC, CD34-PE, and CD45-PerCP-Cy5.5 antibodies (1:200; BD Biosciences), using Ghost Dye Violet (1:1000; Tonbo Biosciences, San Diego, CA, USA) to remove dead cells. Isotype controls were prepared as negative controls. The percentage of antigen-positive cells was evaluated using a FACSVerse II system (BD Biosciences).

### Differentiation assays

For adipogenesis, 100 pretreated human synovial MSCs were plated in 10 cm dishes (Thermo Fisher Scientific) and cultured for 14 days to form colonies. The adherent cells were then cultured for an additional 21 days in an adipogenic induction medium consisting of growth medium supplemented with 100 nM dexamethasone (Wako), 0.5 mM isobutylmethylxanthine (Sigma-Aldrich), 50 mM indomethacin (Sigma-Aldrich), 4.5 mg/mL D-(+)-glucose (Wako), and 10 µg/mL recombinant human insulin (Wako). The cells were then fixed with 10% neutral buffered formalin and stained with Oil Red O (Sigma-Aldrich), and the Oil Red O-positive colonies were counted manually. Each dish was then stained with 0.5% crystal violet to determine the percentage of oil red O-positive colonies.

Osteogenesis assays were performed by plating 100 pretreated MSCs in 10 cm dishes and culturing for 14 days to form colonies. Adherent cells were then cultured in an osteogenic induction medium consisting of growth medium supplemented with 50 μg/mL ascorbic acid 2-phosphate (Wako), 1 nM dexamethasone, and 10 mM β-glycerophosphate (Sigma-Aldrich). After 21 days, the cells were fixed with 10% neutral buffered formalin and stained with Alizarin Red (Sigma-Aldrich). The Alizarin Red-positive colonies were counted manually. Each dish was then stained with 0.5% crystal violet to determine the percentage of Alizarin Red-positive colonies. The intensity of the Alizarin Red staining was calculated using Fiji/Image J.

For chondrogenesis, 2.5 × 10^5^ pretreated MSCs were transferred to a 15 mL polypropylene tube (Thermo Fisher Scientific) and centrifuged at 580 × *g *for 10 min. The pelleted cells were then cultured in chondrogenic induction medium consisting of high glucose Dulbecco’s Modified Eagle Medium (Thermo Fisher Scientific), 1% insulin–transferrin–selenium (ITS; BD Biosciences), 50 µg/mL ascorbate-2-phosphate, 40 µg/mL L-proline (Sigma-Aldrich), 100 nM dexamethasone, 100 µg/mL pyruvate (Sigma-Aldrich), 1% antibiotic–antimycotic, 10 ng/mL transforming growth factor-β3 (Miltenyi Biotec, Bergisch Gladbach, Germany), and 500 ng/mL bone morphogenetic protein-2 (Medtronic, Minneapolis, MN, USA). After 3 weeks of cultivation in a chondrogenic induction medium, 5–6 pellets in each group were weighed. Two pellets each were fixed in 10% neutral buffered formalin, embedded in paraffin, and cut into 5 µm thick sections. The sections were stained with Safranin O (Chroma Gesellschaft Schmid & Co., Munster, Germany) and fast green (Wako) to evaluate the production of glycosaminoglycan (GAG). Three or four pellets from each donor were used in both groups for biochemical analysis.

### Biochemical analysis

GAG and deoxyribonucleic acid (DNA) were quantified by digesting the pellets with 100 µg/mL papain (Sigma-Aldrich) at 65 °C for 16 h. DNA content was measured using Hoechst 33,258 dye (Dojindo, Tokyo, Japan). Fluorescence intensity was measured with a microplate reader (Tecan, Männedorf, Switzerland) at an excitation wavelength of 360 nm, and an emission wavelength of 465 nm. Calf thymus DNA (Sigma-Aldrich) was used to generate a standard curve. The GAG content was then quantified with a Blyscan Kit (Biocolor, Westbury, NY, USA) according to the manufacturer’s instructions. The optical density was measured at 656 nm with a microplate reader, and the total GAG content was calculated. For comparison of the GAG-producing ability, the GAG content was normalized to the DNA content (GAG/DNA). Each experiment was performed in duplicate.

### Immunohistochemistry

Immunostaining of p16, p21, MMP-13, and IL-6 was conducted by immersing paraffin-embedded sections from cartilage pellets in 10 mM Tris containing 1 mM EDTA (pH 9.0) and heating at 95 °C for 1 h to retrieve antigens. For type I, II, and X collagen, the sections were treated with proteinase K (Dako) for 15 min at room temperature. For type II collagen, an additional antigen retrieval step was carried out by incubation with 5 mg/mL hyaluronidase (Sigma) for 1 h at room temperature. The slides were immersed in methanol containing 0.3% H_2_O_2_ for 30 min and then washed with Tris-buffered saline containing 0.1% Tween-20 (TBS-T buffer). After blocking with 5% normal goat serum, the sections were incubated overnight at 4 °C with antibody against p16 (1:200; Abcam), p21 (1:200; Abcam), MMP-13 (1:200; Abcam), IL-6 (1:200; Abcam), type I collagen (1:200; Rockland, Philadelphia, PA, USA), type II collagen (1:50; Santa Cruz Biotechnology, Dallas, TX, USA), and type X collagen (1:500; Sigma-Aldrich). After three washes with TBS-T, the sections were incubated with secondary antibodies conjugated with horseradish peroxidase (1:200; Abcam) for 1 h at room temperature. DAB solution (Dako) was then applied for 5 min, and the cells were counterstained with hematoxylin. DAB and hematoxylin-positive areas in pellets were quantified using the Colour Deconvolution plugin for Fiji/Image J. We normalized the DAB-positive areas to the hematoxylin-positive areas for the evaluation of p16, p21, MMP-13, and IL-6.

### Statistical analysis

Statistical analysis was performed using SPSS (IBM Corp., Chicago, IL, USA). Paired t tests were used for comparison between two groups. One-way analysis of variance, followed by Tukey’s multiple comparisons test, was used for comparison between multiple groups. All *p* values were one-sided, and a *p* value < 0.05 was considered statistically significant.

## Results

### ABT-263 pretreatment decreases the proportion of SA-β-gal-positive cells

**Fig. 2:**
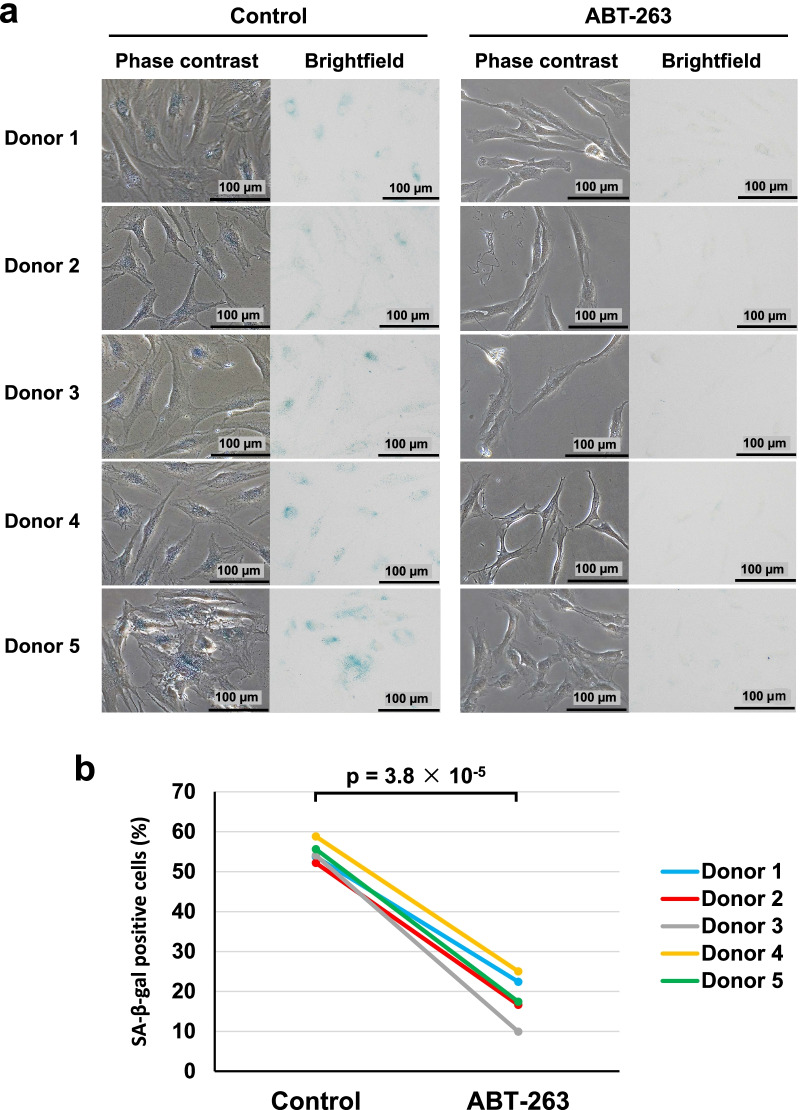
Senescence-associated beta-galactosidase (SA-β-gal) staining. **a** Phase contrast and brightfield images. **b** The ratio of SA-β-gal-positive cells in control and ABT-263 group

An overview of our experimental design is shown in Fig. 1. We pretreated OA synovial MSCs with 0.1% DMSO or 20 µM ABT-263 for 1 day and then performed SA-β-gal staining to evaluate the proportions of senescent cells. The optimal concentration of ABT-263 had been determined in a preliminary study using cells from one donor (Additional file 1: Fig. S1). Phase contrast and brightfield images showed the presence of flattened and enlarged cells in the untreated control group, and those cells showed positive staining for SA-β-gal staining (Fig. 2a). By contrast, the culture treated with ABT-263 showed few flattened and enlarged cells, and the percentage of SA-β-gal-positive cells was significantly lower than in the control group (Fig. 2b, *p* = 3.8 × 10^–5^). The average percentages of senescence cells in the control and ABT-263 groups were 54.8% and 18.2%, respectively.

We also used western blotting to determine the activity of BCL-2 after pretreatment with ABT-263. The protein expression levels of BCL-2 were significantly lower in the ABT-263 group than in the control group (Additional file 1: Fig. S2, *p* = 0.028).

### ABT-263 induces apoptosis in senescent cells

**Fig. 3:**
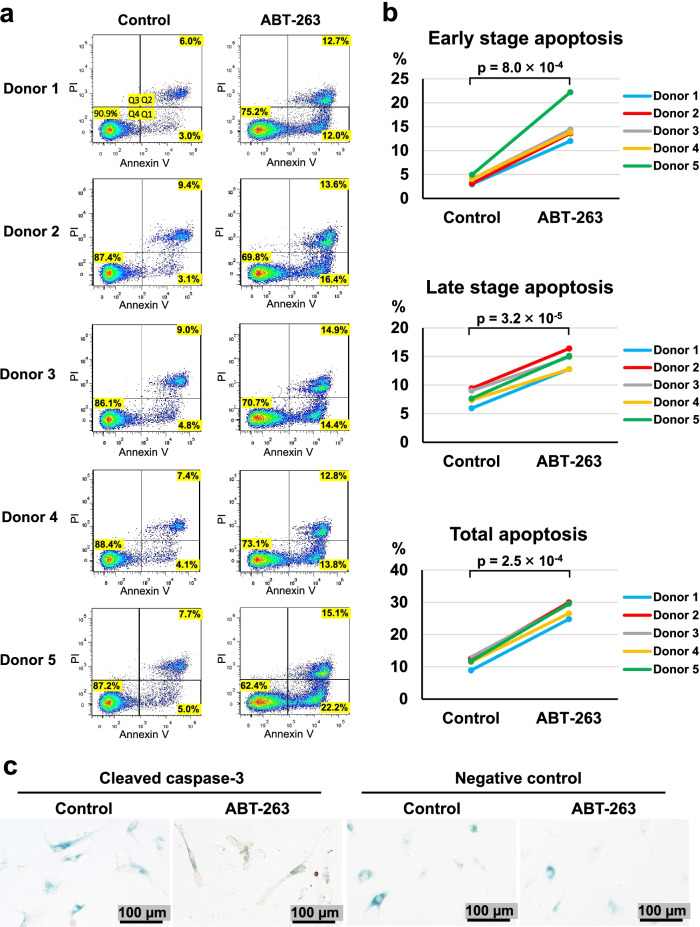
Apoptosis assay. **a** Flow cytometry analysis of annexin V/propidium iodide (PI). Dot plots show the percentages of viable (Q4), early apoptotic (Q1), and late apoptotic (Q2) cells. **b** The ratio of early, late, and total apoptotic cells in the control and ABT-263 groups. **c** Co-staining of senescence-associated beta-galactosidase (blue) and cleaved caspase-3 (brown) after the treatment with ABT-263

We confirmed whether ABT-263 induces apoptosis in senescent cells by assessing early- and late-stage apoptosis by annexin V/propidium iodide (PI) staining in control OA synovial MSCs and in OA synovial MSCs treated with ABT-263 (Fig. 3a). The MSCs treated with ABT-263 underwent significantly greater levels of early-stage apoptosis (*p* = 8.0 × 10^–4^), late-stage apoptosis (*p* = 3.2 × 10^–5^), and total apoptosis (*p* = 2.5 × 10^–4^) (Fig. 3b). Co-staining for SA-β-gal and cleaved caspase-3, a molecule with a known pivotal role in the apoptotic cascade [27], revealed no staining for caspase-3 in the SA-β-gal-positive cells of the control group, whereas the cells in the ABT-263 group were positive for cleaved caspase-3 (Fig. 3c).

### ABT-263 pretreatment increases the numbers and sizes of colonies

**Fig. 4:**
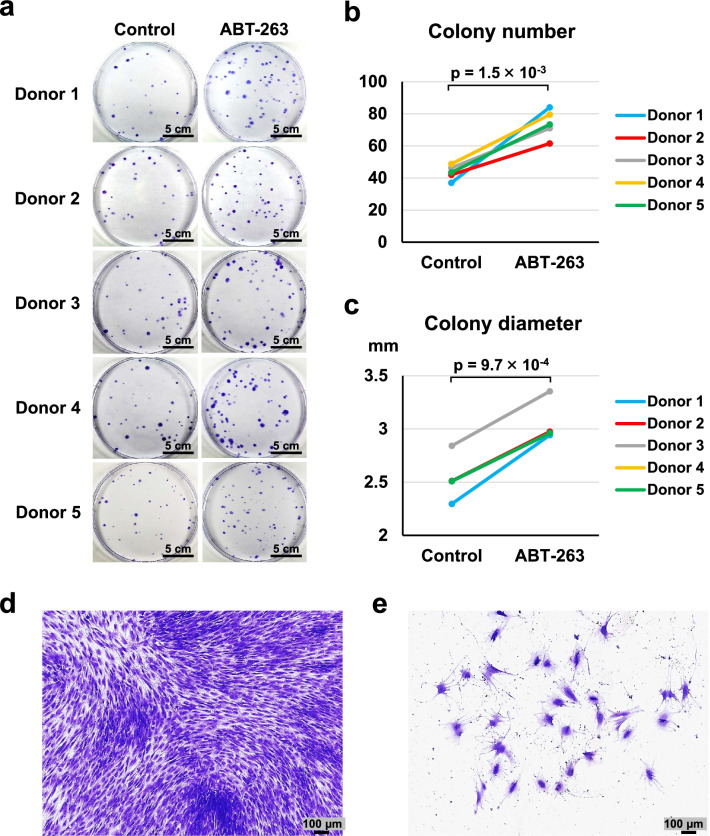
Colony-forming ability. **a** Representative images of colonies stained with crystal violet (4 replicate dishes per donor). **b** Colony numbers per dish in the control and ABT-263 groups. **c** Diameter of colonies in the control and ABT-263 groups. **d** Representative image of colony-forming cells. **e** Representative image of flattened and enlarged cells that did not form colonies

We examined the effect of ABT-263 pretreatment on self-renewal and proliferation properties by conducting colony-forming assays on 100 pretreated cells cultured for 14 days. Representative images of the colonies stained with crystal violet are shown in Fig. 4a. The colony numbers were significantly greater in the ABT-263 group than in the control group (Fig. 4b, *p* = 1.5 × 10^–3^). The colony diameters were also significantly larger in the ABT-263 group than in the control group (Fig. 4c, *p* = 9.7 × 10^–4^). Histogram distributions of the colony diameters showed a shift toward larger colonies in response to ABT-263 pretreatment (Additional file 1: Fig. S3). Most colony-forming cells were small and spindle-shaped, both in the control and the ABT-263 groups (Fig. 4d). However, the few flattened and enlarged cells that did not form colonies were observed predominantly in the control group (Fig. 4e).

### ABT-263 pretreatment affects the expression of surface antigens

**Fig. 5:**
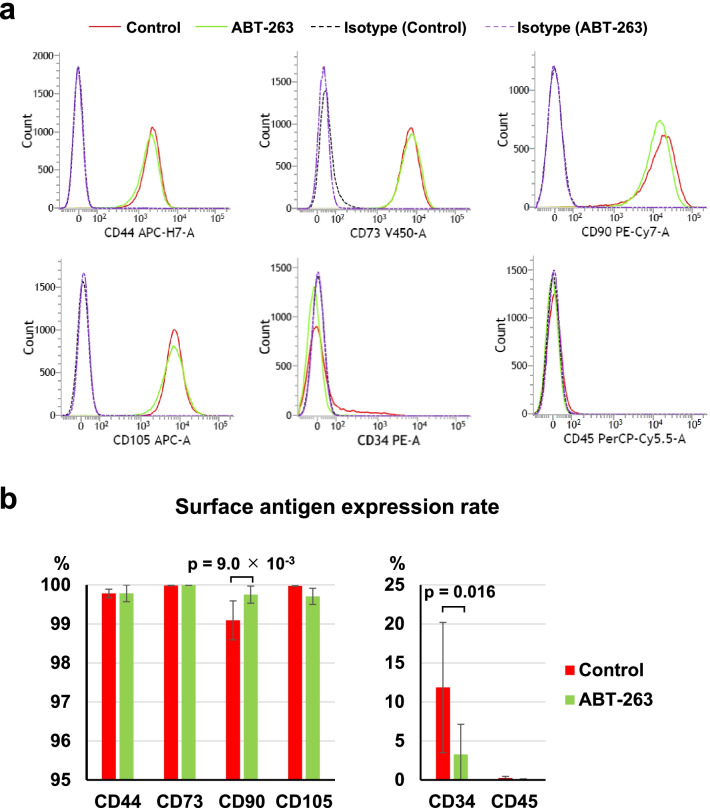
Surface antigen expression. **a** Representative flow cytometry histograms. Red solid lines indicate the control group, and green solid lines indicate the ABT-263 group. Dotted lines show the isotype control. **b** The expression rate of each surface antigen. Values are shown as mean ± SD from 5 donors

We used flow cytometry to assess the effect of ABT-263 pretreatment on surface antigen expression. The analyses demonstrated an almost 100% mean rate of positivity for MSC markers such as CD44, CD73, CD90, and CD105 in both the control and ABT-263 groups (Fig. 5a), but the difference in the CD90 positive rate between the control and ABT-263 groups was statistically significant (*p* = 9.0 × 10^–3^) (Fig. 5b). The mean rates of positivity for CD34 were 11.9% in the control group and 3.3% in the ABT-263 group. The positive rate for CD34 was significantly lower in the ABT-263 group than in the control group (Fig. 5b, Additional file 1: Fig. S4, *p* = 0.016). The mean rates of positivity for CD45 were 0.2% in the control group and 0.1% in the ABT-263 group.

### ABT-263 pretreatment enhances adipogenic potential

**Fig. 6:**
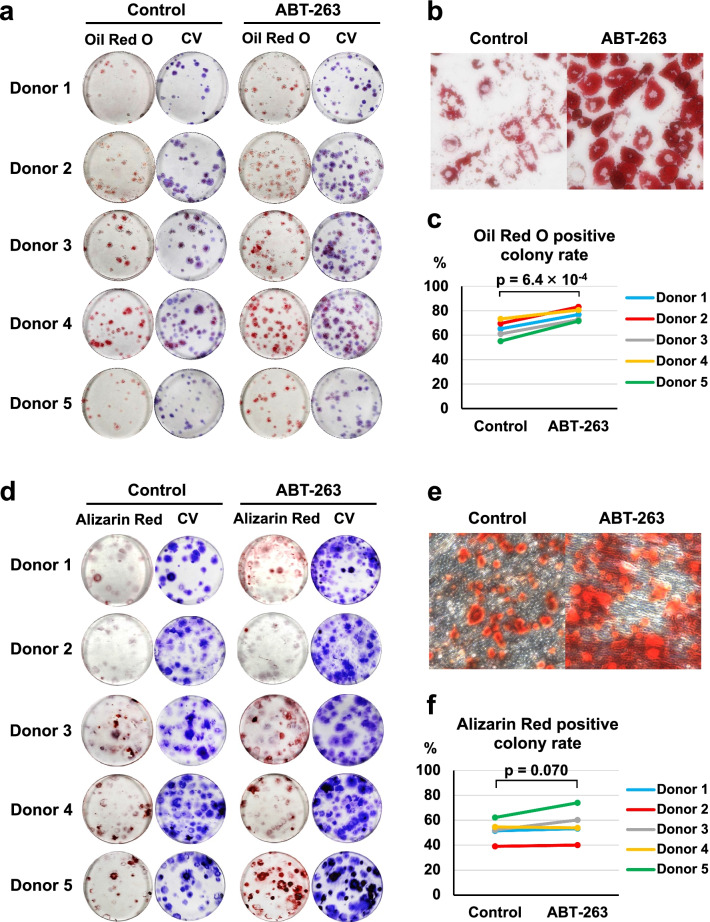
Adipogenic and osteogenic differentiation. **a** Representative images of Oil Red O and crystal violet (CV) staining (3 replicate dishes per donor). **b** Reperesentative microscopic images of Oil Red O staining. **c** The percentage of Oil Red O-stained colonies in the control and ABT-263 groups. **d** Representative images of Alizarin Red and CV staining (3 replicate dishes per donor). **e** Reperesentative microscopic images of Alizarin Red staining. **f** The percentage of Alizarin Red-stained colonies in the control and ABT-263 groups

We investigated the effect of ABT-263 pretreatment on adipogenesis and osteogenesis in single-cell-derived colonies by culturing 100 pretreated cells in growth medium for 14 days to allow colony formation, followed by culture in induction medium for 21 days. Macroscopic images of Oil Red O and subsequent crystal violet staining are shown in Fig. 6a. Observation by light microscopy confirmed that pretreatment with ABT-263 enhanced Oil Red O staining, indicating increased lipid production by the cells (Fig. 6b). Quantitative analysis showed that the percentage of Oil Red O-stained colonies was significantly higher in the ABT-263 group than in the control group (Fig. 6c, *p* = 6.4 × 10^–4^).

Macroscopic images of the cells stained with Alizarin Red and subsequent crystal violet staining are shown in Fig. 6d. The cells in both the control and the ABT-63 groups underwent calcification (Fig. 6e). No significant difference was detected in the proportion of Alizarin Red-stained colonies between the control and ABT-263 groups (Fig. 6f, *p* = 0.070). No significant difference was detected in the intensity of Alizarin Red staining between the control and ABT-263 groups (Additional file 1: Fig. S5).

### ABT-263 pretreatment enhances chondrogenic potential

**Fig. 7:**
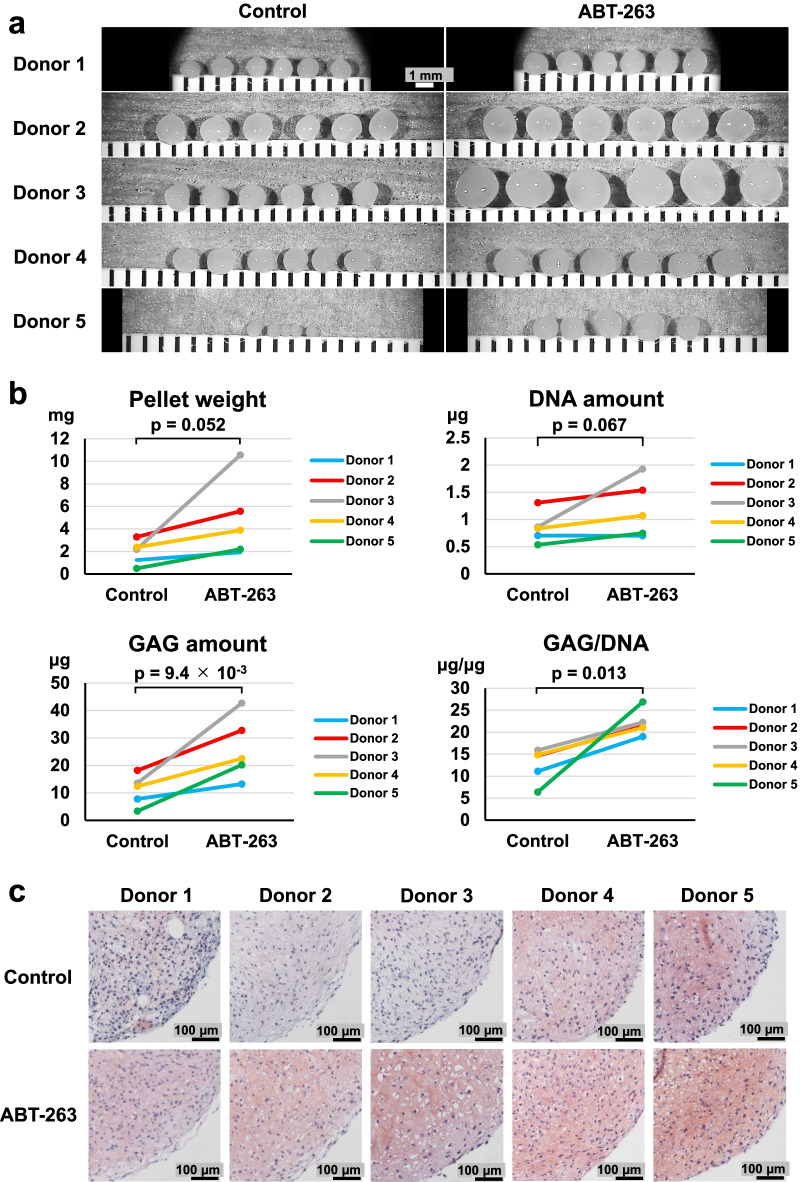
Chondrogenic differentiation. **a** Gross appearance of chondrogenic pellets (5 or 6 replicate pellets per donor). **b** Pellet wet weight, DNA quantification, glycosaminoglycan (GAG) quantification, and GAG/DNA ratio (3 or 4 replicate pellets per donor). **c** Representative images of Safranin O staining of chondrogenic pellets

We assessed the chondrogenic potential of ABT-263-treated cells by culturing pretreated cells for 21 days in chondrogenic induction medium. The cells formed larger pellets after chondrogenic induction when pretreated with ABT-263 compared to the control cells (Fig. 7a). The mean pellet wet weight from the control and ABT-263 groups was 1.9 mg and 4.8 mg, respectively, confirming a greater pellet mass in the ABT-263 group than in the control group (*p* = 0.052) (Fig. 7b).

Biochemical analyses revealed significantly increased GAG amounts (*p* = 9.4 × 10^–3^) and a higher GAG/DNA ratio (*p* = 0.013) in the ABT-263 group than in the control group; however, no significant difference was found in the DNA amounts between the two groups (Fig. 7b). Safranin O staining was more intense in the pellets from the ABT-263 group than from the control group (Fig. 7c). The percentage of type I collagen positive areas was significantly lower in the ABT-263 group than in the control group (Additional file 1: Fig. S6a, *p* = 9.3 × 10^–3^). The percentage of type II collagen positive areas was significantly higher in the ABT-263 group than in the control group (Additional file 1: Fig. S6b, *p* = 0.042). Type X collagen was not detected in either group (Additional file 1 Fig. S6c).

### ABT-263 pretreatment decreased SASP expression in chondrogenic pellets

**Fig. 8:**
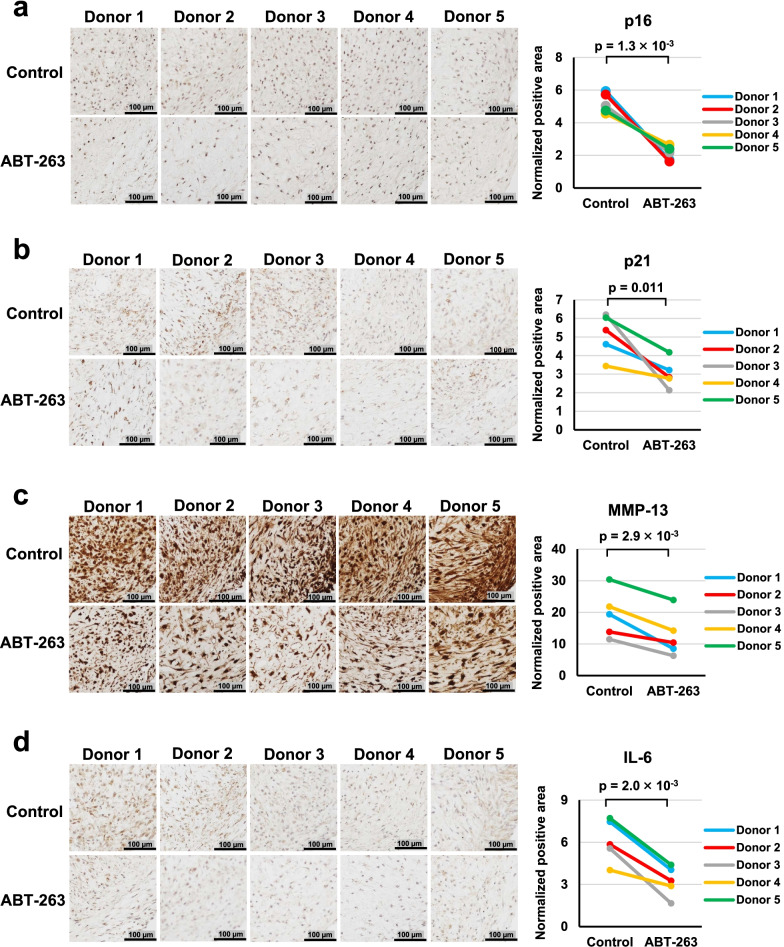
Immunohistochemistry of senescence markers in chondrogenic pellets. Representative images and positive ratios of **a** p16, **b** p21, **c** MMP-13, and **d** IL-6 immunostaining in the control and ABT-263 groups

We examined the effect of ABT-263 pretreatment on the expression of senescence-related markers during chondrogenesis by immunostaining chondrogenic pellets for senescence markers (p16 and p21) and SASP markers (MMP-13 and IL-6). Representative images and the ratios of the stained areas are shown in Fig. 8. The normalized positive areas for p16, p21, MMP-13, and IL-6 were significantly lower in the ABT-263 group than in the control group (*p* = 1.3 × 10^–3^, 0.011, 2.9 × 10^–3^, and 2.0 × 10^–3^, respectively).

## Discussion

In the present study, we demonstrated that senescent cells showing positive staining for SA-β-gal in human synovial MSC samples obtained from OA patients were effectively removed by pretreatment with 20 µM ABT-263, leading to decreased BCL-2 expression. Our apoptosis assay revealed that ABT-263 treatment induced both early- and late-stage apoptosis in the senescent OA synovial MSCs. In addition, the SA-β-gal-positive cells expressed cleaved caspase-3 after the treatment. These results indicate that ABT-263 could selectively kill senescent cells present in OA synovial MSC samples by inducing apoptosis. Previous studies have shown that senescent cells in human chondrocyte and bone marrow-derived MSC samples showed dose-dependent and statistically significantly decreases in the numbers of SA-β-gal-positive cells when treated with ABT-263 [26, 28]. Similar effects of ABT-263 were also observed in our study.

Our colony formation assays indicated that the number and diameter of colonies were significantly greater in the ABT-263 group than in the untreated control group. The control group also contained a few colonies with flattened and enlarged cells, indicative of senescent cells, which are generally growth-arrested and have enlarged and flattened morphologies [29, 30]. Therefore, our results suggested that senescent cells that have poor colony-forming abilities were eliminated by the ABT-263 treatment, leaving MSCs with high colony formation potential in the final culture.

Most cells in both groups showed positive immunostaining for CD44, CD73, CD90, and CD105, despite the successful clearing of senescent cells by the ABT-263 treatment. Consequently, none of these can be considered a candidate for use as a specific marker for senescent cells. On the contrary, the mean positivity for CD34 (MSC negative marker) was significantly decreased by ABT-263 treatment, from 11.9% in the control group to 3.3% in the ABT-263 group. A previous report showed that CD34-positive synovial fibroblasts from OA and rheumatoid arthritis tissues release high levels of inflammatory cytokines [31]. Similar characteristics are also observed in senescent cells; therefore, investigating the relationship between CD34 expression and cellular senescence in the synovium would be an interesting future study.

In terms of multipotency, we noted a significant improvement in adipogenic potential in cells treated with ABT-263, but the osteogenic potential did not improve. Wagner et al. demonstrated a deterioration in adipogenic potential in human MSCs with increasing passage number, but did not see an equivalent decline in osteogenesis [32]. However, Bertolo et al. reported opposite findings [33]. Therefore, no consensus exists regarding the adipogenic and osteogenic capacities of senescent MSCs. Regarding chondrogenesis, we observed that the synovial MSCs pretreated with ABT-263 formed cartilaginous pellets rich in GAGs and type II collagen and showed decreased expression of type I collagen, senescence markers, and SASP factors. Therefore, these cells are expected to induce better cartilage regeneration than untreated cells by producing more hyaline cartilage matrix and releasing fewer of the catabolic factors that deteriorate the functions of the surrounding cells. Our study had one significant limitation, as we did not investigate the in vivo therapeutic effect of ABT-263-treated MSCs. Further research using animal models of cartilage/meniscus injury or OA is needed to verify our results. Another limitation of our study was the lack of quantification (e.g., by ELISA) of the released SASP factors.

MSCs are a heterogeneous population, so purification is sometimes carried out to isolate true stem cells [34]. MSCs are typically purified by fluorescence-activated cell sorting (FACS) [35], but the lack of specific surface markers for senescent cells limits this approach [36]. We propose that the purification of MSCs could be improved by administration of a senolytic agent, such as ABT-263. Care would be needed when using ABT-263 in terms of cytotoxic effects; however, when compared with FACS, ABT-263 pretreatment is easy, inexpensive, and requires no special equipment. Senescence of various MSC types is also known to be accelerated during aging of individuals and during in vitro serial passaging of MSCs in culture [32, 37]. ABT-263 pretreatment could also be beneficial in these cases.

## Conclusions

Pretreatment with ABT-263 selectively eliminated senescent cells in synovial MSCs derived from OA patients. The pretreated MSCs consistently showed higher colony formation, adipogenic, and chondrogenic abilities, and decreased SASP expression. These results indicate that ABT-263 could be a useful senolytic drug to improve the function of synovial MSCs obtained from OA patients and could hold promise for the development of effective cell-based OA therapy.

## Supplementary Information

**Additional file 1. Fig. S1.** Determination of the optimal concentration of ABT-263. (**a**) Synovial mesenchymal stem cells from one donor were pretreated with 0, 5, 10, 15, or 20 µM ABT-263, and stained with SA-β-gal. Brightfield and phase contrast images are shown. (**b**) The percentage of SA-β-gal positive cells pretreated with 0, 5, 10, 15, and 20 μM ABT-263. The experiment was performed in triplicate wells. **Fig. S2.** Western blot analysis of BCL-2. (**a**) Protein bands of BCL-2 and β-actin in the control and ABT-263 group. (**b**) BCL-2 expression was normalized to the respective β-actin expression. **Fig. S3.** Histograms of colony diameters. Blue bars show the control group and red bars show the ABT-263 group. The overlay histograms on the right show the distributions for each donor. Pooled data from all donors was shown at the bottom. Histogram distribution shifted towards larger colonies by ABT-263 pretreatment. **Fig. S4.** Flow cytometry dot plots for CD34. **Fig. S5.** Relative intensity of Alizarin Red staining. **Fig. S6.** Immunohistochemistry of collagen. (**a**) Representative images and positive ratios of (**a**) type I collagen, (**b**) type II collagen, and (**c**) type X collagen in the control and ABT-263 groups.

## Data Availability

The datasets generated and analyzed during the current study are available from the corresponding author on reasonable request.

## Abbreviations

α-MEM: α-Modified essential medium
BCL-2: B cell lymphoma 2
DAB: Diaminobenzidine
DMSO: Dimethyl sulfoxide
FACS: Fluorescence-activated cell sorting
GAG: Glycosaminoglycan
ITS: Insulin–transferrin–selenium
IL-6: Interleukin-6
MRI: Magnetic resonance imaging
MMP-13: Matrix metalloprotease-13
MSCs: Mesenchymal stem cells
OA: Osteoarthritis
PBS: Phosphate-buffered saline
PI: Propidium iodide
SASP: Senescence-associated secretory phenotype
SA-β-gal: Senescence-associated β-galactosidase

## References

[1] Jeon OH, David N, Campisi J, Elisseeff JH (2018). Senescent cells and osteoarthritis: a painful connection. J Clin Invest.

[2] Sellam J, Berenbaum F (2010). The role of synovitis in pathophysiology and clinical symptoms of osteoarthritis. Nat Rev Rheumatol.

[3] Taruc-Uy RL, Lynch SA (2013). Diagnosis and treatment of osteoarthritis. Prim Care.

[4] Bianco P, Robey PG, Simmons PJ (2008). Mesenchymal stem cells: revisiting history, concepts, and assays. Cell Stem Cell.

[5] Barry F (2019). MSC therapy for osteoarthritis: an unfinished story. J Orthop Res.

[6] Li M, Luo X, Lv X, Liu V, Zhao G, Zhang X (2016). In vivo human adipose-derived mesenchymal stem cell tracking after intra-articular delivery in a rat osteoarthritis model. Stem Cell Res Ther.

[7] Satué M, Schüler C, Ginner N, Erben RG (2019). Intra-articularly injected mesenchymal stem cells promote cartilage regeneration, but do not permanently engraft in distant organs. Sci Rep.

[8] Harada Y, Nakasa T, Mahmoud EE, Kamei G, Adachi N, Deie M (2015). Combination therapy with intra-articular injection of mesenchymal stem cells and articulated joint distraction for repair of a chronic osteochondral defect in the rabbit. J Orthop Res.

[9] Sakaguchi Y, Sekiya I, Yagishita K, Muneta T (2005). Comparison of human stem cells derived from various mesenchymal tissues: superiority of synovium as a cell source. Arthritis Rheum.

[10] Yoshimura H, Muneta T, Nimura A, Yokoyama A, Koga H, Sekiya I (2007). Comparison of rat mesenchymal stem cells derived from bone marrow, synovium, periosteum, adipose tissue, and muscle. Cell Tissue Res.

[11] Mochizuki T, Muneta T, Sakaguchi Y, Nimura A, Yokoyama A, Koga H (2006). Higher chondrogenic potential of fibrous synovium- and adipose synovium-derived cells compared with subcutaneous fat-derived cells: distinguishing properties of mesenchymal stem cells in humans. Arthritis Rheum.

[12] De Bari C, Dell'Accio F, Tylzanowski P, Luyten FP (2001). Multipotent mesenchymal stem cells from adult human synovial membrane. Arthritis Rheum.

[13] Koga H, Muneta T, Nagase T, Nimura A, Ju YJ, Mochizuki T (2008). Comparison of mesenchymal tissues-derived stem cells for in vivo chondrogenesis: suitable conditions for cell therapy of cartilage defects in rabbit. Cell Tissue Res.

[14] Horie M, Driscoll MD, Sampson HW, Sekiya I, Caroom CT, Prockop DJ (2012). Implantation of allogenic synovial stem cells promotes meniscal regeneration in a rabbit meniscal defect model. J Bone Jt Surg Am.

[15] Ozeki N, Muneta T, Koga H, Nakagawa Y, Mizuno M, Tsuji K (2016). Not single but periodic injections of synovial mesenchymal stem cells maintain viable cells in knees and inhibit osteoarthritis progression in rats. Osteoarthr Cartil.

[16] Sekiya I, Katano H, Mizuno M, Koga H, Masumoto J, Tomita M (2021). Alterations in cartilage quantification before and after injections of mesenchymal stem cells into osteoarthritic knees. Sci Rep.

[17] van Deursen JM (2014). The role of senescent cells in ageing. Nature.

[18] Jeon OH, Kim C, Laberge RM, Demaria M, Rathod S, Vasserot AP (2017). Local clearance of senescent cells attenuates the development of post-traumatic osteoarthritis and creates a pro-regenerative environment. Nat Med.

[19] Del Rey MJ, Valín Á, Usategui A, Ergueta S, Martín E, Municio C (2019). Senescent synovial fibroblasts accumulate prematurely inrheumatoid arthritis tissues and display an enhanced inflammatory phenotype. Immun Ageing.

[20] Huang J, Chen C, Liang C, Luo P, Xia G, Zhang L (2020). Dysregulation of the Wnt signaling pathway and synovial stem cell dysfunction in osteoarthritis development. Stem Cells Dev.

[21] Kirkland JL, Tchkonia T (2020). Senolytic drugs: from discovery to translation. J Intern Med.

[22] Zhu Y, Tchkonia T, Fuhrmann-Stroissnigg H, Dai HM, Ling YY, Stout MB (2016). Identification of a novel senolytic agent, navitoclax, targeting the Bcl-2 family of anti-apoptotic factors. Aging Cell.

[23] Zhu Y, Tchkonia T, Pirtskhalava T, Gower AC, Ding H, Giorgadze N (2015). The Achilles' heel of senescent cells: from transcriptome to senolytic drugs. Aging Cell.

[24] Chang J, Wang Y, Shao L, Laberge RM, Demaria M, Campisi J (2016). Clearance of senescent cells by ABT263 rejuvenates aged hematopoietic stem cells in mice. Nat Med.

[25] Zhang L, Pitcher LE, Prahalad V, Niedernhofer LJ, Robbins PD (2022). Targeting cellular senescence with senotherapeutics: senolytics and senomorphics. FEBS J.

[26] Grezella C, Fernandez-Rebollo E, Franzen J, Ventura Ferreira MS, Beier F, Wagner W (2018). Effects of senolytic drugs on human mesenchymal stromal cells. Stem Cell Res Ther.

[27] Gown AM, Willingham MC (2002). Improved detection of apoptotic cells in archival paraffin sections: immunohistochemistry using antibodies to cleaved caspase 3. J Histochem Cytochem.

[28] Yang H, Chen C, Chen H, Duan X, Li J, Zhou Y (2020). Navitoclax (ABT263) reduces inflammation and promotes chondrogenic phenotype by clearing senescent osteoarthritic chondrocytes in osteoarthritis. Aging (Albany NY).

[29] Hayflick L, Moorhead PS (1961). The serial cultivation of human diploid cell strains. Exp Cell Res.

[30] Shirasawa S, Sekiya I, Sakaguchi Y, Yagishita K, Ichinose S, Muneta T (2006). In vitro chondrogenesis of human synovium-derived mesenchymal stem cells: optimal condition and comparison with bone marrow-derived cells. J Cell Biochem.

[31] Mizoguchi F, Slowikowski K, Wei K, Marshall JL, Rao DA, Chang SK (2018). Functionally distinct disease-associated fibroblast subsets in rheumatoid arthritis. Nat Commun.

[32] Wagner W, Horn P, Castoldi M, Diehlmann A, Bork S, Saffrich R (2008). Replicative senescence of mesenchymal stem cells: a continuous and organized process. PLoS ONE.

[33] Bertolo A, Baur M, Guerrero J, Pötzel T, Stoyanov J (2019). Autofluorescence is a reliable in vitro marker of cellular senescence in human mesenchymal stromal cells. Sci Rep.

[34] Zha K, Li X, Yang Z, Tian G, Sun Z, Sui X (2021). Heterogeneity of mesenchymal stem cells in cartilage regeneration: from characterization to application. NPJ Regen Med.

[35] Gullo F, De Bari C (2013). Prospective purification of a subpopulation of human synovial mesenchymal stem cells with enhanced chondro-osteogenic potency. Rheumatology (Oxford).

[36] Scudellari M (2017). To stay young, kill zombie cells. Nature.

[37] Kapetanos K, Asimakopoulos D, Christodoulou N, Vogt A, Khan W (2021). Chronological age affects MSC senescence in vitro: a systematic review. Int J Mol Sci.

