# Supplementary material for: Clearance of senescent cells with ABT-263 improves biological functions of synovial mesenchymal stem cells from osteoarthritis patients
Source: Stem Cell Res Ther. 2022 Jun 3;13:222. doi: 10.1186/s13287-022-02901-4 (PMC9166575; doi:10.1186/s13287-022-02901-4)
Supplement: Supplementary file 1 — Additional file 1. Fig. S1. Determination of the optimal concentration of ABT-263. (a) Synovial mesenchymal stem cells from one donor were pretreated with 0, 5, 10, 15, or 20 µM ABT-263, and stained with SA-β-gal. Brightfield and phase contrast images are shown. (b) The percentage of SA-β-gal positive cells pretreated with 0, 5, 10, 15, and 20 μM ABT-263. The experiment was performed in triplicate wells. Fig. S2. Western blot analysis of BCL-2. (a) Protein bands of BCL-2 and β-actin in the control and ABT-263 group. (b) BCL-2 expression was normalized to the respective β-actin expression. Fig. S3. Histograms of colony diameters. Blue bars show the control group and red bars show the ABT-263 group. The overlay histograms on the right show the distributions for each donor. Pooled data from all donors was shown at the bottom. Histogram distribution shifted towards larger colonies by ABT-263 pretreatment. Fig. S4. Flow cytometry dot plots for CD34. Fig. S5. Relative intensity of Alizarin Red staining. Fig. S6. Immunohistochemistry of collagen. (a) Representative images and positive ratios of (a) type I collagen, (b) type II collagen, and (c) type X collagen in the control and ABT-263 groups. [file 13287_2022_2901_MOESM1_ESM.pptx]

## Slide 1
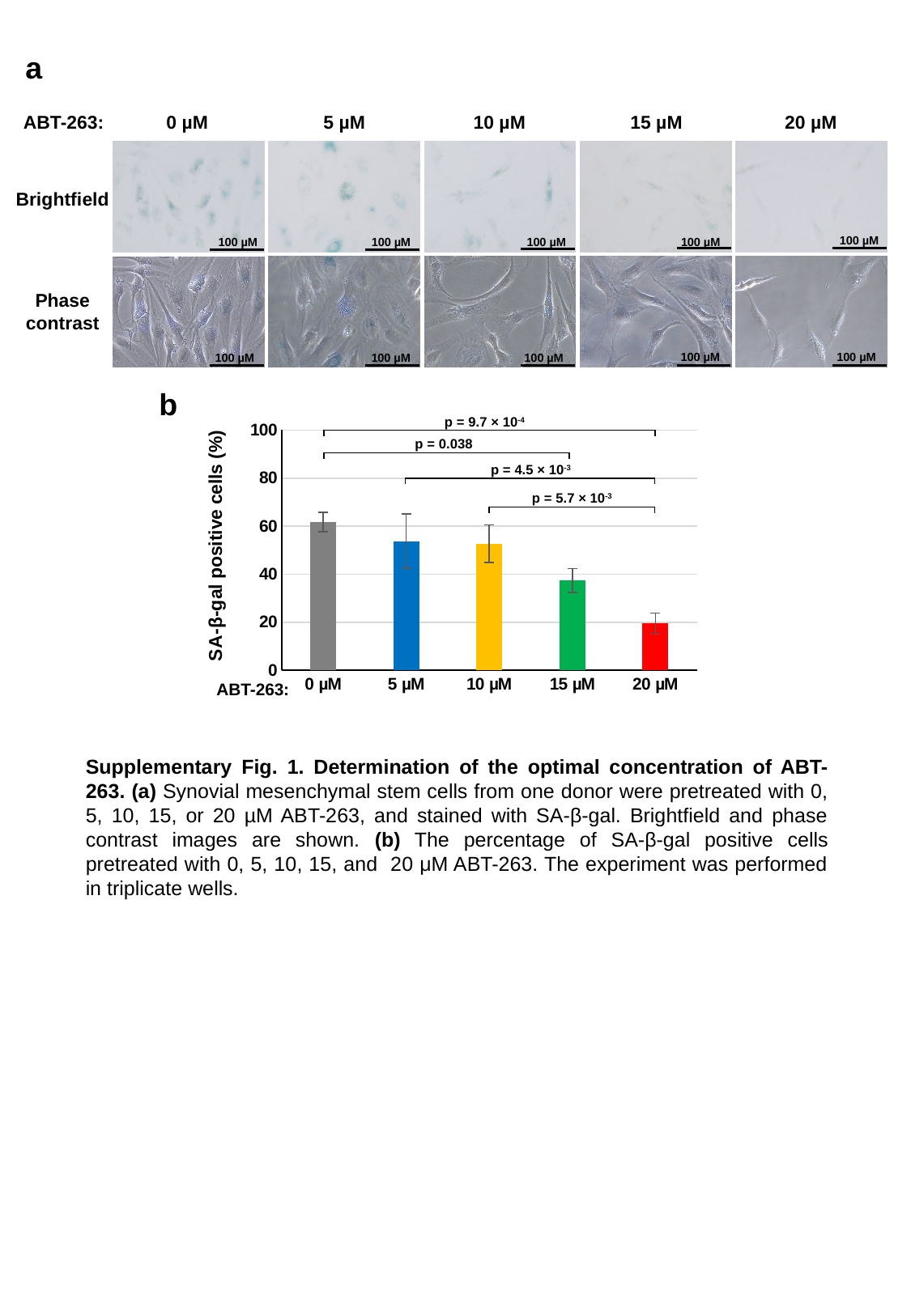

a
ABT-263:
15 µM
5 µM
10 µM
20 µM
0 µM
Brightfield
100 µM
100 µM
100 µM
100 µM
100 µM
Phase contrast
100 µM
100 µM
100 µM
100 µM
100 µM
b
### Chart
| Category | |
|---|---|
| 0 µM | 61.70956136709561 |
| 5 µM | 53.811303027742994 |
| 10 µM | 52.681159420289845 |
| 15 µM | 37.327512669978425 |
| 20 µM | 19.453825307483843 |
p = 9.7 × 10^-4^
p = 0.038
p = 4.5 × 10^-3^
p = 5.7 × 10^-3^
ABT-263:
Supplementary Fig. 1. Determination of the optimal concentration of ABT-263. (a) Synovial mesenchymal stem cells from one donor were pretreated with 0, 5, 10, 15, or 20 µM ABT-263, and stained with SA-β-gal. Brightfield and phase contrast images are shown. (b) The percentage of SA-β-gal positive cells pretreated with 0, 5, 10, 15, and 20 μM ABT-263. The experiment was performed in triplicate wells.

## Slide 2
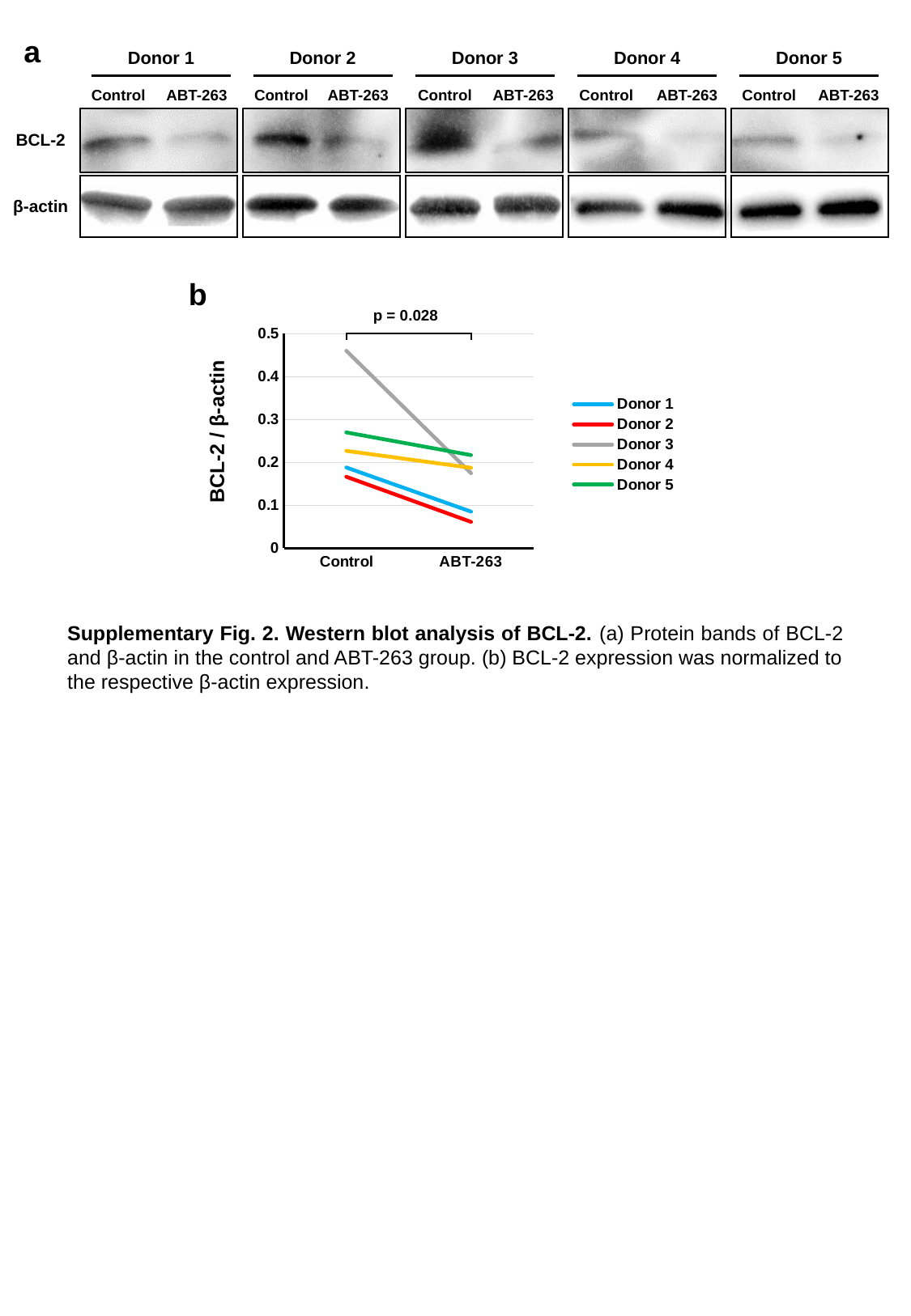

a
Donor 1
Donor 2
Donor 3
Donor 4
Donor 5
Control
ABT-263
Control
ABT-263
Control
ABT-263
Control
ABT-263
Control
ABT-263
BCL-2
β-actin
b
p = 0.028
### Chart
| Category | | | | | |
|---|---|---|---|---|---|
| Control | 0.1882023082419582 | 0.16665930982517896 | 0.46029987880414425 | 0.2269959307954544 | 0.2700516216793065 |
| ABT-263 | 0.08546559158881221 | 0.0615135036943138 | 0.17484312319727274 | 0.18749976976491464 | 0.2170552949723329 |BCL-2 / β-actin
Supplementary Fig. 2. Western blot analysis of BCL-2. (a) Protein bands of BCL-2 and β-actin in the control and ABT-263 group. (b) BCL-2 expression was normalized to the respective β-actin expression.

## Slide 3
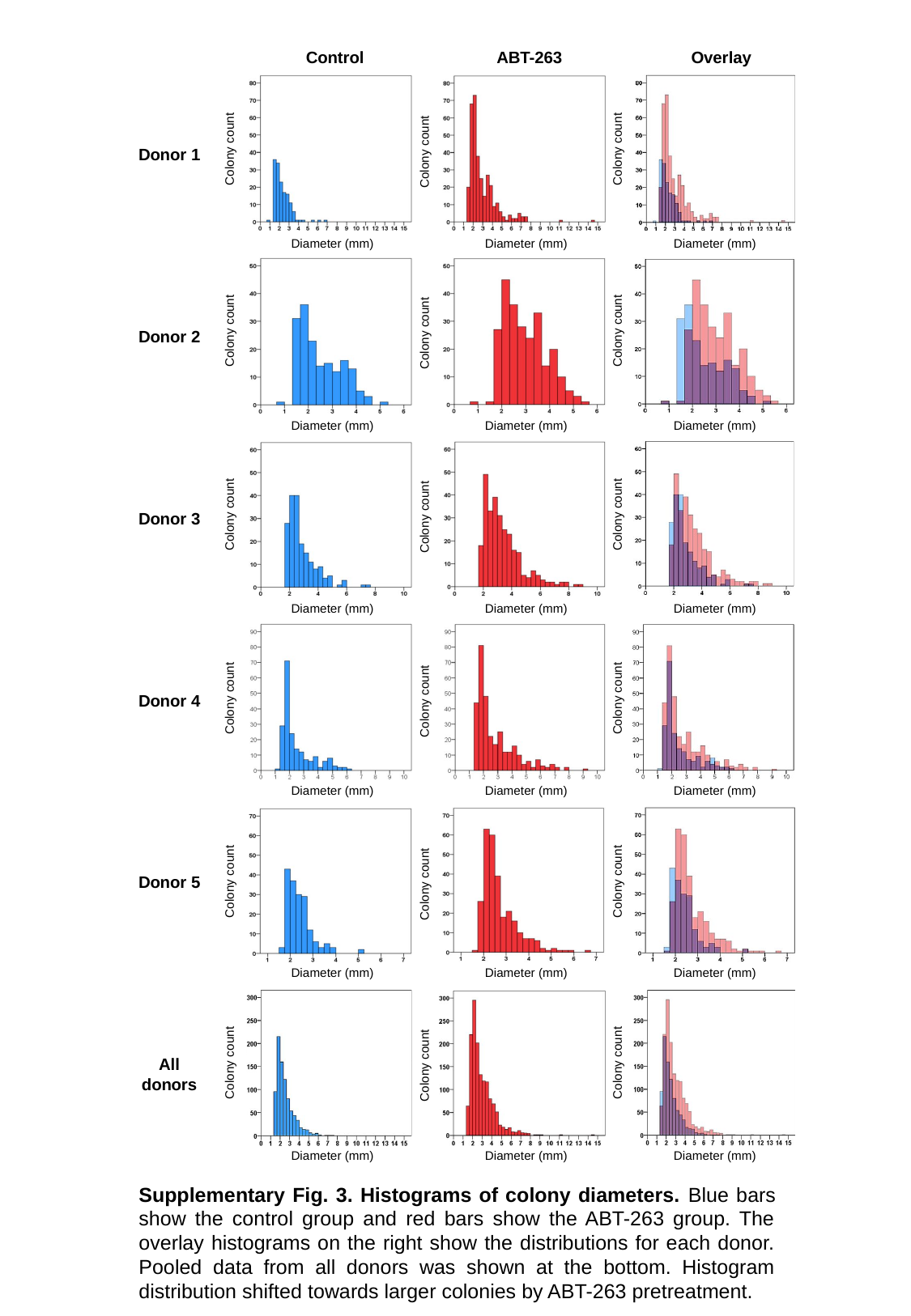

Control
ABT-263
Overlay
Colony count
Colony count
Colony count
Donor 1
Diameter (mm)
Diameter (mm)
Diameter (mm)
Colony count
Colony count
Colony count
Donor 2
Diameter (mm)
Diameter (mm)
Diameter (mm)
Colony count
Colony count
Colony count
Donor 3
Diameter (mm)
Diameter (mm)
Diameter (mm)
Colony count
Colony count
Colony count
Donor 4
Diameter (mm)
Diameter (mm)
Diameter (mm)
Colony count
Colony count
Colony count
Donor 5
Diameter (mm)
Diameter (mm)
Diameter (mm)
Colony count
Colony count
Colony count
All donors
Diameter (mm)
Diameter (mm)
Diameter (mm)
Supplementary Fig. 3. Histograms of colony diameters. Blue bars show the control group and red bars show the ABT-263 group. The overlay histograms on the right show the distributions for each donor. Pooled data from all donors was shown at the bottom. Histogram distribution shifted towards larger colonies by ABT-263 pretreatment.

## Slide 4
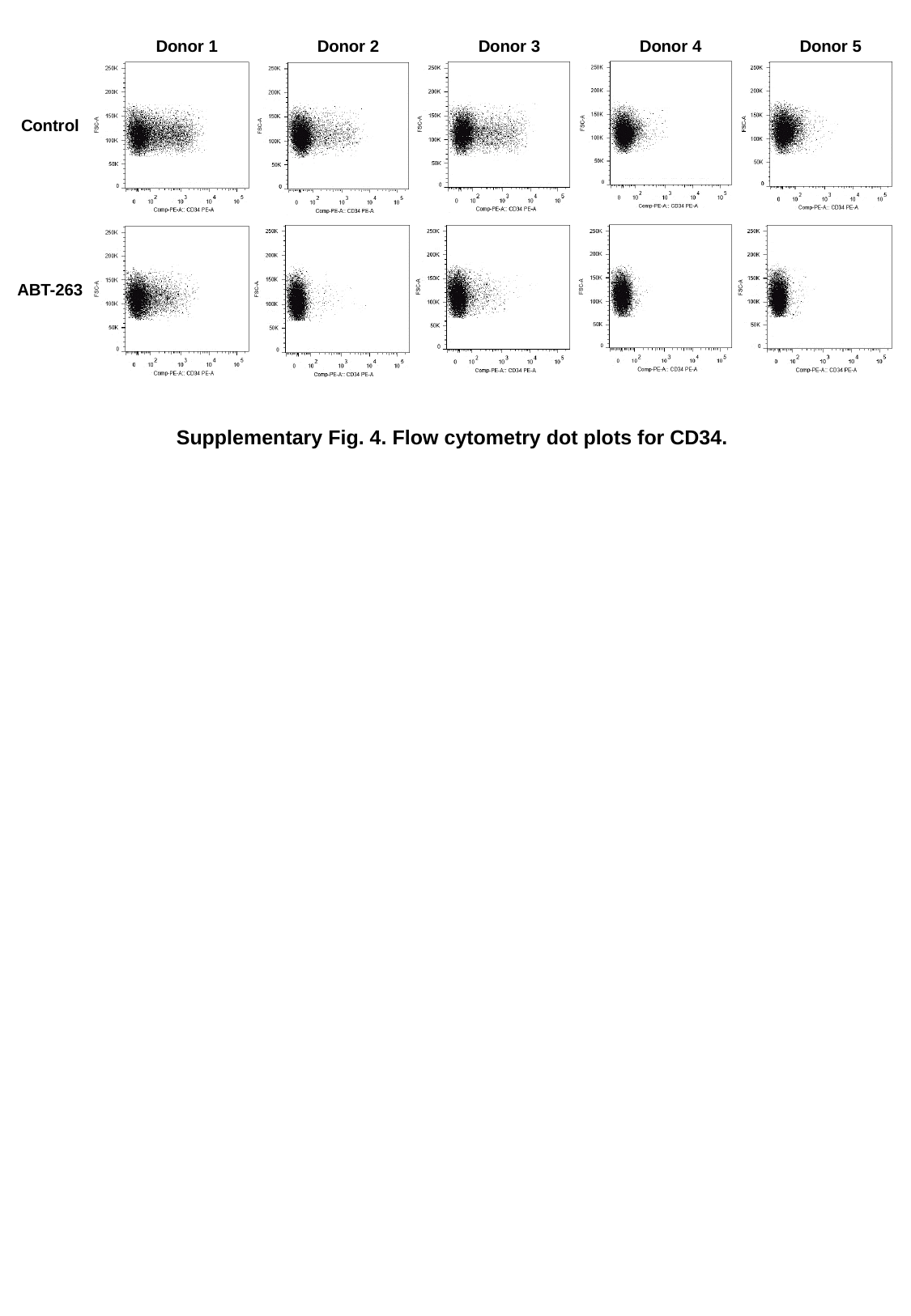

Donor 1
Donor 2
Donor 3
Donor 4
Donor 5
Control
ABT-263
Supplementary Fig. 4. Flow cytometry dot plots for CD34.

## Slide 5
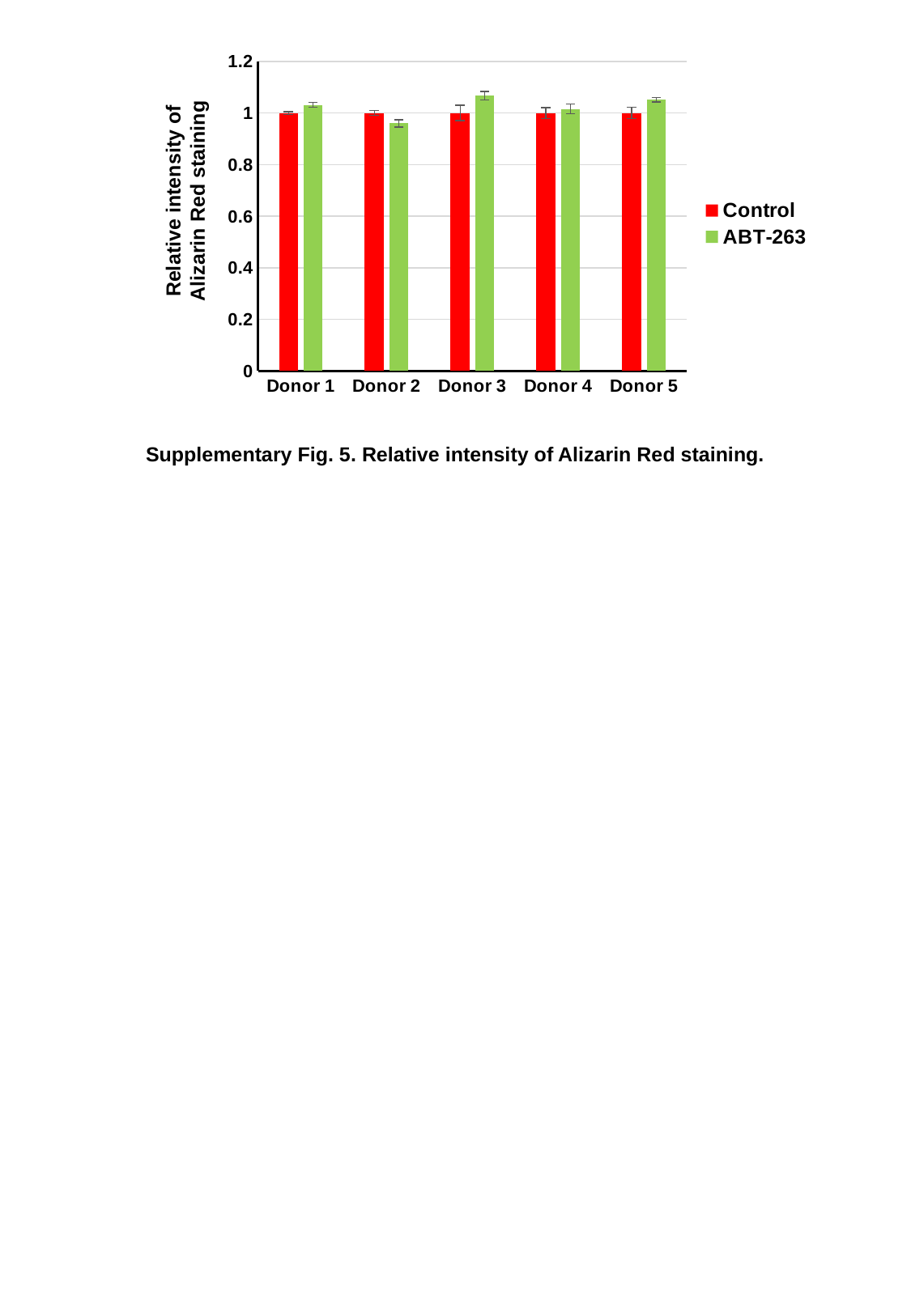

### Chart
| Category | | |
|---|---|---|
| Donor 1 | 1.0 | 1.0316126590096326 |
| Donor 2 | 1.0 | 0.9599745121848589 |
| Donor 3 | 1.0 | 1.0667815446155666 |
| Donor 4 | 1.0 | 1.0155869773158681 |
| Donor 5 | 1.0 | 1.051580801814691 |Relative intensity of Alizarin Red staining
Supplementary Fig. 5. Relative intensity of Alizarin Red staining.

## Slide 6
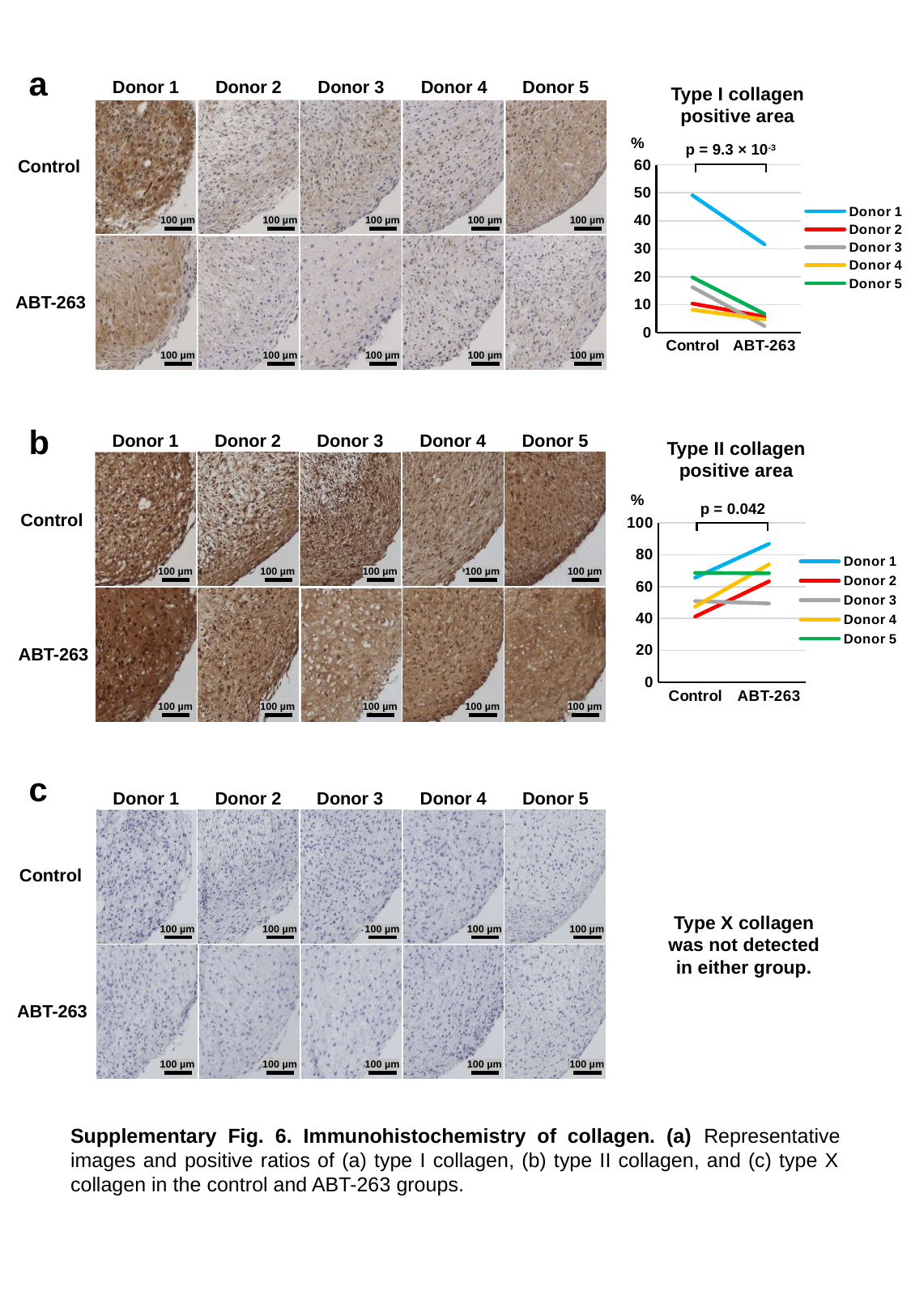

a
Donor 1
Donor 2
Donor 3
Donor 4
Donor 5
Type I collagen positive area
%
p = 9.3 × 10^-3^
Control
### Chart
| Category | | | | | |
|---|---|---|---|---|---|
| Control | 49.107 | 10.386 | 16.241 | 8.161 | 19.771 |
| ABT-263 | 31.568768722018724 | 5.682709516691578 | 2.3613789861884444 | 4.717456721046245 | 6.663921365638767 |100 µm
100 µm
100 µm
100 µm
100 µm
ABT-263
100 µm
100 µm
100 µm
100 µm
100 µm
b
Donor 1
Donor 2
Donor 3
Donor 4
Donor 5
Type II collagen positive area
%
### Chart
| Category | | | | | |
|---|---|---|---|---|---|
| Control | 65.591 | 41.143 | 50.898 | 47.448 | 68.546 |
| ABT-263 | 86.81988636622933 | 63.29591928574867 | 49.39148044624747 | 73.95518918918917 | 68.37856186918859 |
p = 0.042
Control
100 µm
100 µm
100 µm
100 µm
100 µm
ABT-263
100 µm
100 µm
100 µm
100 µm
100 µm
c
Donor 1
Donor 2
Donor 3
Donor 4
Donor 5
Control
Type X collagen was not detected in either group.
100 µm
100 µm
100 µm
100 µm
100 µm
ABT-263
100 µm
100 µm
100 µm
100 µm
100 µm
Supplementary Fig. 6. Immunohistochemistry of collagen. (a) Representative images and positive ratios of (a) type I collagen, (b) type II collagen, and (c) type X collagen in the control and ABT-263 groups.
